# Energetically relevant predator–prey body mass ratios and their relationship with predator body size

**DOI:** 10.1002/ece3.4715

**Published:** 2018-12-27

**Authors:** Jonathan C. P. Reum, Kirstin K. Holsman, Kerim Y. Aydin, Julia L. Blanchard, Simon Jennings

**Affiliations:** ^1^ School of Aquatic and Fishery Sciences University of Washington Seattle Seattle Washington; ^2^ Alaska Fisheries Science Center National Marine Fisheries Service, NOAA Seattle Washington; ^3^ Institute for Marine and Antarctic Studies and Centre for Marine Socioecology University of Tasmania Hobart Tasmania Australia; ^4^ International Council for the Exploration of the Sea København V Denmark

**Keywords:** body size, ecosystem, food web, piscivory, size spectrum, trophic level

## Abstract

Food web structure and dynamics depend on relationships between body sizes of predators and their prey. Species‐based and community‐wide estimates of preferred and realized predator–prey mass ratios (PPMR) are required inputs to size‐based size spectrum models of marine communities, food webs, and ecosystems. Here, we clarify differences between PPMR definitions in different size spectrum models, in particular differences between PPMR measurements weighting prey abundance in individual predators by biomass (*r* ^bio^) and numbers (*r* ^num^). We argue that the former weighting generates PPMR as usually conceptualized in equilibrium (static) size spectrum models while the latter usually applies to dynamic models. We use diet information from 170,689 individuals of 34 species of fish in Alaskan marine ecosystems to calculate both PPMR metrics. Using hierarchical models, we examine how explained variance in these metrics changed with predator body size, predator taxonomic resolution, and spatial resolution. In the hierarchical analysis, variance in both metrics emerged primarily at the species level and substantially less variance was associated with other (higher) taxonomic levels or with spatial resolution. This suggests that changes in species composition are the main drivers of community‐wide mean PPMR. At all levels of analysis, relationships between weighted mean *r* ^bio^ or weighted mean *r* ^num^ and predator mass tended to be dome‐shaped. Weighted mean *r* ^num^ values, for species and community‐wide, were approximately an order of magnitude higher than weighted mean *r* ^bio^, reflecting the consistent numeric dominance of small prey in predator diets. As well as increasing understanding of the drivers of variation in PPMR and providing estimates of PPMR in the north Pacific Ocean, our results demonstrate that that *r* ^bio^ or *r* ^num^, as well as their corresponding weighted means for any defined group of predators, are not directly substitutable. When developing equilibrium size‐based models based on bulk energy flux or comparing PPMR estimates derived from the relationship between body mass and trophic level with those based on diet analysis, weighted mean *r* ^bio^ is a more appropriate measure of PPMR. When calibrating preference PPMR in dynamic size spectrum models then weighted mean *r* ^num^ will be a more appropriate measure of PPMR.

## 1 INTRODUCTION

Body size is the principle factor structuring biomass, numerical abundances, trophic levels, and predator–prey interactions in marine and freshwater ecosystems (Dickie, Kerr, & Boudreau, 1987; Trebilco, Baum, Salomon, & Dulvy, 2013). In most instances, predators feed on smaller‐bodied prey (Barnes, Maxwell, Reuman, & Jennings, 2010; Brose, Jonsson, et al., 2006; Cohen, Pimm, Yodzis, & Saldana, 1993). Predator‐to‐prey body mass ratios (PPMR) are particularly relevant for understanding regularities in the size structuring of predator–prey interactions in food webs and can vary based on individual‐ or species‐level attributes of predators (Barnes et al., 2010; Brose, Jonsson, et al., 2006; Nakazawa, Ushio, & Kondoh, 2011; Reum & Hunsicker, 2012). Importantly, food web structure and dynamics, as represented in a variety of size‐based modeling frameworks, are sensitive to the PPMR of predators (e.g., Brose, Williams, & Martinez, 2006; Law, Plank, & Kolding, 2016; Otto, Rall, & Brose, 2007), which suggests PPMR offers a useful metric for functionally characterizing predators.

Size spectra describe the abundance of individuals in a food web as a function of body size (Sheldon, Prakash, & Sutcliffe, 1972). The first size spectrum models were developed to explain remarkably consistent size spectra slopes in pelagic food webs (Sheldon et al., 1972; Sprules & Barth, 2015), with recent extensions developed to investigate human and environmental impacts on marine ecosystems (e.g., Blanchard et al., 2014; Jacobsen, Burgess, & Andersen, 2017; Jennings & Blanchard, 2004; Jennings & Collingridge, 2015; Rochet & Benoît, 2012). Broadly, size spectrum models can be divided according to whether they provide equilibrium (static) predictions of size spectra or model system processes and size distributions dynamically (Blanchard, Heneghan, Everett, Trebilco, & Richardson, 2017). The two approaches, however, conceptualize PPMR differently, with implications for how PPMR should be calculated from empirical diet data.

A central premise in all size spectrum models is that the size of prey consumed is linked to the size of predators, although specifics of implementation vary among models (Andersen, Jacobsen, & Farnsworth, 2016; Blanchard et al., 2017; Guiet, Poggiale, & Maury, 2016). In dynamic size spectrum models, predation is modeled mechanistically and within a given time increment is either fully or partly a function of prey densities and the prey size preference of the predator (Benoît & Rochet, 2004; Hartvig, Andersen, & Beyer, 2011). Prey size preferences are usually modeled using a log‐normal selectivity function, or feeding kernel (Andersen et al., 2016). Prey mass at the peak of the feeding kernel is defined by a “preferred PPMR” parameter, which reflects the behaviorally and morphologically mediated prey choice of the predator when presented with prey of many sizes, and a second parameter controls the feeding kernel width (Andersen et al., 2016). The “realized PPMR” (i.e., PPMR based on ingested prey) of predators is emergent in the models and may change with predator size and prey relative abundance (Hartvig et al., 2011). Direct estimation of preferred PPMR is challenging because this requires knowledge of realized PPMR and the size composition and abundance of encountered prey (Floeter & Temming, 2003; Tsai, Hsieh, & Nakazawa, 2016; Ursin, 1973, 1974). Alternatively, it may be possible to approximate preferred PPMR with a simple offset from realized PPMR. For instance, simulation studies suggest preferred PPMR may be ~60% of mean realized PPMR (Hartvig et al., 2011). This approximation has been used to estimate preferred PPMR from diet‐based estimates of realized mean PPMR for species in multispecies size spectrum models calibrated to real ecosystems (Blanchard et al., 2014).

In static size spectra models, species identity is ignored and aggregate community biomass is indexed by body size (Blanchard et al., 2017). The models define PPMR as a realized community‐wide mean that is constant across predator sizes. Consequently, PPMR sets the prey size class that supports production in a given predator size class (e.g., Borgmann, 1987; Sheldon, Sutcliffe, & Paranjape, 1977; Thiebaux & Dickie, 1992; Thiebaux & Dickie, 1993). Since these models characterize the transfer of energy from prey to predators, empirical estimates of realized community‐wide mean PPMR need to account for the energetic contribution of differently sized prey to predator diets. Estimates of realized community‐wide mean PPMRs, which reflect the energetic contribution of prey to predator diets, have been estimated from community‐wide relationships between body mass and trophic level with nitrogen stable isotope methods (Al‐Habsi, Sweeting, Polunin, & Graham, 2008; Jennings & Blanchard, 2004; Jennings & Mackinson, 2003; Jennings, Pinnegar, Polunin, & Boon, 2001; Jennings, Pinnegar, Polunin, & Warr, 2002; Reum, Jennings, & Hunsicker, 2015) and have been used to parameterize equilibrium size spectrum models (Jennings & Blanchard, 2004). Realized community‐wide mean PPMR influences food chain length, transfer efficiency, and size spectrum slopes (Jennings & Warr, 2003; Jennings et al., 2001; Jennings, Warr, & Mackinson, 2002). The few available empirical estimates of realized community‐wide mean PPMR are based on stable isotope analyses rather than diet data, largely because PPMR estimates of individual predator–prey events (i.e., “individual‐link PPMR”; Nakazawa et al., 2011) are rarely available for all species in a community due to the intensive sampling required.

Existing studies of PPMR based on diet data have focused on analyzing patterns in individual‐link PPMR (e.g., Barnes et al., 2010; Brose, Jonsson, et al., 2006; Klecka & Boukal, 2013; Nakazawa et al., 2011; Reum & Hunsicker, 2012) which is related to realized PPMR in dynamic size spectrum models (Hartvig et al., 2011; Tsai et al., 2016). At the level of an individual predator *i*, the realized mean PPMR ($r_{i}^{\mathrm{num}}$) is:

(1) $$r_{i}^{\mathrm{num}}=\frac{1}{n}\sum_{j=1}^{n}\frac{M_{i}}{m_{j}}$$

where *M* is the body mass of the predator and *m* is the body mass of individual *j* = 1, 2, …, *n* prey observed in the predator stomach. The mean of $r_{i}^{\mathrm{num}}$ for any defined group of individual predators is the mean of the $r_{i}^{\mathrm{num}}$ values for all predators in the group weighted by the relative abundance of prey observed in each individual predator.

The dependence of $r_{i}^{\mathrm{num}}$ on prey numerical abundance, coupled with the higher numbers of small relative to large prey in size‐based food webs (Trebilco et al., 2013), implies that $r_{i}^{\mathrm{num}}$ does not reflect the contribution of different sizes of prey to the energy intake of a predator and is therefore less appropriate for generating estimates of realized community‐wide mean PPMR in static size spectrum models. This would be addressed by recognizing prey contributions to diet in terms of biomass ($r_{i}^{\mathrm{bio}}$), where $r_{i}^{\mathrm{bio}}$ is calculated as follows:

(2) $$r_{i}^{\mathrm{bio}}=\sum_{j=1}^{n}\frac{M_{i}}{m_{j}}\times \frac{m_{i}}{w_{i}}$$

where *w* is the total biomass of all prey recovered from predator *i.* Here, energy and mass are assumed to be related by a mass–caloric conversion factor and are regarded as equivalent (e.g., Thiebaux & Dickie, 1993). An equivalent expression of Equation (2) is simply the predator mass divided by the average body mass of individual prey (i.e., “individual predator PPMR,” Nakazawa et al., 2011). That is,

(3) $$r_{i}^{\mathrm{bio}}=\frac{M_{i}}{\frac{1}{n}\sum_{j=1}^{n}m_{j}}$$

While $r_{i}^{\mathrm{num}}$ is the average of individual‐link PPMRs, $r_{i}^{\mathrm{bio}}$ is the ratio between predator mass and the average prey mass. To arrive at an estimate of mean PPMR that reflects the energetic contribution of differently sized prey for a group of individual predators, the constituent $r_{i}^{\mathrm{bio}}$ values need to be weighted in a manner that accounts for differences in the relative total biomass of prey in the individual predators.

To compare $r_{i}^{\mathrm{bio}}$ and $r_{i}^{\mathrm{num}}$, consider a 1,000‐g predator with stomach contents comprising two fish of 25 g and two krill of 0.1 g. The corresponding $r_{i}^{\mathrm{bio}}$ and $r_{i}^{\mathrm{num}}$ values will be 79 and 5,020, respectively, with $r_{i}^{\mathrm{bio}}$ heavily weighted downward by the larger prey. This general pattern also holds for the weighted means of $r_{i}^{\mathrm{bio}}$ and $r_{i}^{\mathrm{num}}$ for predators within a given group. The measures convey different but complementary information, but $r_{i}^{\mathrm{bio}}$ has received considerably less attention in diet‐based studies of PPMR.

Here, we use diet data for 34 species of fish predators from Alaskan marine ecosystems (Livingston et al., 2017) to estimate mean *r* ^num^ and mean *r* ^bio^. Specifically, we used hierarchical models to examine how mean *r* ^num^ and mean *r* ^bio^ changes with predator body mass. Dynamic size spectrum models suggest that mean *r* ^num^ should exhibit an overall positive increase with predator body mass and a secondary, nonlinear scaling due to oscillations in the relative abundances of small and large‐bodied prey (Hartvig et al., 2011). The models predict oscillatory behavior in the scaling of biomass with body mass, whereby traveling waves propagate down the size spectrum, reflecting the growth of individuals into successively larger size classes (Law, Plank, & James, 2009). The relative encounter rates of small and large‐bodied prey within the feeding kernel of predators changes with predator size, resulting in nonlinear patterns in community‐wide mean *R* ^num^ with body mass (Hartvig et al., 2011). In cross‐system studies using empirical diet data, individual‐link PPMR appears to increase linearly with predator body sizes on log–log scales (Barnes et al., 2010; Brose, Jonsson, et al., 2006; Nakazawa et al., 2011) and nonlinearity, while tested for infrequently, has been observed in one intensively sampled food web (Reum & Hunsicker, 2012). In addition, we evaluated how predator taxonomic resolution and spatial resolution account for variance in mean *r* ^num^ and mean *r* ^bio^. Previous analyses have shown considerable variation in individual‐link PPMR across taxonomic groupings (Naisbit, Kehrli, Rohr, & Bersier, 2011; Nakazawa et al., 2011), but variation with spatial scale has received little attention. We use the fitted hierarchical model to produce a preliminary estimate of community‐wide mean *r* ^bio^ to compare with realized community‐wide mean *r* ^num^ and describe implications for food web analysis and size‐based food web modeling.

## 2 MATERIALS AND METHODS

### 2.1 Diet data

Diet data used in this study described the stomach contents of fish collected during the NOAA Alaska Fisheries Science Center (AFSC) groundfish trawl surveys. The surveys have been conducted annually in the Eastern Bering Sea (EBS) since 1979 and biennially or triennially around the Aleutian Islands (AI) and in the Gulf of Alaska (GoA) since 1993 and 1984, respectively (Livingston et al., 2017). At each station, fish brought on board were sorted according to species and sex, weighed, enumerated, and individuals were measured for length to the nearest cm to enable estimation of population size structure within survey strata in each region. The number of individuals sampled for length for a species was dependent on the size range of that species in the haul, up to a maximum of 300 individuals (for details see Stauffer, 2004).

Species selected for stomach contents analysis varied interannually. “Core” commercial species, including walleye pollock, Pacific cod, arrowtooth flounder, Pacific halibut in all three ecosystems, and Pacific Ocean perch and Atka mackerel in the GoA and AI, are sampled in every survey. Three to five non‐core species are sampled in each survey on a rotating basis, with the aim of rotating through all commercial or ecologically important species over a 5‐year period (Livingston et al., 2017). Individuals chosen for stomach content analysis were selected to span a wide body length range given the available fish (Livingston et al., 2017). After they have been individually weighed and measured their stomach contents are preserved in 10% buffered formalin for subsequent processing in the laboratory. All sampling is performed from May to September, with most individuals (92%) sampled in June, July, and August.

In the laboratory, the stomach contents of each individual predator were sorted to the lowest possible taxonomic level and by life history stage, and in most cases were individually weighed and measured. Prey digestion level, based on a visual assessment of the percentage of intact prey body mass, was also recorded (Livingston et al., 2017). Large numbers of smaller prey (e.g., copepods, amphipods, euphausiids) were not always weighed and measured individually, and aggregate weights and counts were recorded. The diet data described are available through an online database maintained by NOAA Alaska Fisheries Science Center (https://access.afsc.noaa.gov/REEM/WebDietData/DietDataIntro.php). Additional details on the survey methods and diet collection protocols are available elsewhere (Livingston et al., 2017).

Records of prey that were largely digested (<75% intact) were excluded. However, when length data were available for digested individual fish and crab prey, the corresponding undigested mass was estimated using species‐ and life history‐specific length–weight relationships estimated from weight and length measurements of largely undigested prey (>75% intact; JCP Reum, *unpublished data*). If no individual prey body measurements were recorded, they were estimated in one of two ways. First, if total mass and count information were available (64% of records), we calculated mean body mass by dividing the total recorded weight by number of individuals. This approach was predominately applied to data for small‐bodied invertebrates (e.g., copepods, amphipods, euphausiids). Second, if total weight for each prey species and/or life history stage were recorded but count and length information were not (19% of records), we made the simplifying assumption that individual body mass was the same as mean mass calculated from records with both total mass and count. This assumption is similar to those made in other studies of prey size (e.g., Tsai et al., 2016) and was required because there has been little focus in many large‐scale diet studies on acquiring individual body size measurements for small‐bodied prey. Although authors have reasonably cautioned against using mean body sizes of either predators or prey to calculate PPMR (Nakazawa, 2015, 2017; Nakazawa et al., 2011), we believe the benefits outweigh the risks in our analysis because body mass variation in the prey categories for which we had to estimate individual body mass was low (much less than an order of magnitude). Discarding these records would lead to a systematic underestimation of the importance of small‐bodied prey in predator diets (e.g., Jacob et al., 2011).

For predators, individual body mass was not always recorded (46%). In these cases, body mass was estimated using species‐specific length–weight relationships fitted to individual length–weight data from the survey (JCP Reum, *unpublished data*). Once the preceding approaches had been applied to the raw diet data, the data used for this analysis comprised records of individual predator mass, classified by species and the body mass or estimated body mass of the prey recorded in their stomachs, classified by life stage and to the lowest possible taxonomic resolution.

We calculated weighted means of *r* ^num^ and *r* ^bio^ for all predator species within a defined body mass class (log_10_ body mass intervals of 0.1) and subregion within the EBS, AI, or GoA. Records were aggregated at this level because stomach content data are noisy given the partly stochastic nature of prey encounters over short periods of time and because our main goal was to resolve spatial and size‐based shifts in PPMR at the population level. Subregions within the EBS, AI, and GoA were based on ecosystem subregions and fisheries management zones and were used to assess potential spatial variation in PPMR.

Weighted mean *r* ^num^ for all *i = *1, …*x* predators in any defined group (*R* ^num^) was calculated as follows:

(4) $$R^{\mathrm{num}}=\sum_{i=1}^{x}r_{i}^{\mathrm{num}}\times \frac{n_{i}}{N}$$

where *N* is the sum of all prey observed in the group. These estimates of *R* ^num^ thus account for small variations in individual predator body masses within a body mass class and are weighted by the relative number of prey recorded in each individual predator. Weighted mean *r* ^bio^ for all predators in any defined group (*R* ^bio^) was calculated as follows:

(5) $$R^{\mathrm{bio}}=\sum_{i=1}^{x}r_{i}^{\mathrm{bio}}\times p_{i}$$

where

(6) $$p_{i}=\frac{w_{i}/M_{i}}{\sum w_{i}/M_{i}}$$

That is, *p_i_* is the specific total prey mass (g prey g predator^−1^) observed in predator *i* relative to the sum of specific total prey masses observed for all predators in the same group. The weighting based on specific total prey mass standardizes for energetic importance given small variations in individual predator body sizes within the predator body mass classes and extends the same prey biomass weighting approach used for $r_{i}^{\mathrm{bio}}$ (Equation (2)) up to a group‐level mean estimate. The mean predator body mass for individuals in each predator body size class was also calculated as a weighted average (following the same weighting method used for *R* ^num^ or *R* ^bio^) for use as a predictor variable in the statistical analysis. *R* ^num^ or *R* ^bio^ were calculated only for species, size class, and subregions with diet records from a minimum of ten individual predators.

### 2.2 Statistical analysis

*R* ^num^ and *R* ^bio^ were modeled using a linear mixed effects model with a nested random effects grouping structure where subregion was nested in region, region within species, species within families, and families within orders. Because the phylogeny is known for only a subset of predators in the diet data set, taxonomy was used as a proxy for phylogeny (e.g., Naisbit et al., 2011). A nested grouping structure was used to account for taxonomic nonindependence in the data set (e.g., Blackburn & Duncan, 2001; Sunday, Bates, & Dulvy, 2011) and because we sought to explicitly estimate the proportion of variance in *R* ^num^ and *R* ^bio^ associated with each taxonomic level (Reum & Marshall, 2013). The family and order of each predator was obtained from the Integrated Taxonomic Information System (www.itis.gov; accessed March 2017). The fixed effects terms included a linear and quadratic predator body mass predictors. At each model level, variance components corresponding to the intercepts and slopes for the linear and quadratic predictor variables were estimated. The model included a quadratic body mass term to account for potential nonlinear relationship between *R* ^num^ or *R* ^bio^ and predator body mass as suggested by other empirical studies (Reum & Hunsicker, 2012) and theoretical models (Hartvig et al., 2011). Preliminary analyses indicated that centering and scaling the linear and quadratic log_10_‐transformed predator body mass predictor variables obviated the need to estimate the full variance–covariance matrix for the random effects (Zuur, 2009). Consequently, at each level of nesting, the random intercept and slope coefficients for the linear and quadratic predictor variables were assumed uncorrelated and normally distributed.

Prior to model fitting, the response variables (*R* ^num^ and *R* ^bio^) were log_10_‐transformed to better conform to assumptions of normality. The models were fitted under a Bayesian framework using the statistical library “brms” (Bürkner, 2017) for the “R” software program v. 3.3 (R Development Core Team, 2015). The library utilizes the software package “Stan” which employs Hamiltonian Monte Carlo and its extension, No‐U‐Turn Sampler. The algorithms produce samples that are much less autocorrelated and are generally more efficient at reaching convergence than more commonly used algorithms (Bürkner, 2017). For the fixed effects parameters, normal prior distributions were used with a mean and variance of 0 and 3, respectively. A half‐Cauchy prior was placed on the standard deviation of each random effect, with location and scale parameters set to 0 and 10, respectively (Gelman, 2006). A half Student *t* prior was used for the residual variance, with shape and scale parameters equal to 0.001 (Gelman, 2006). Three MCMC chains were run in parallel for 1,550 simulation iterations with a burn‐in of 50 iterations. A thinning interval of 3 was selected to reduce autocorrelation in the posterior draws, resulting in 1,500 posterior distribution samples of the model parameter estimates from which median parameter estimates were calculated, and 95% highest posterior density (HPD) credible intervals were constructed. To ensure convergence, traceplots of the chains and diagnostic values $\left(\sqrt{\hat{R}}\right)$ were visually inspected, where values close to 1 (<1.2) suggest convergence (Gelman et al., 2014). With the fitted models, we examined the relative importance of each level of nesting in terms of prediction improvement. This was performed by evaluating the Bayesian *R* ^2^ or “explained variance” (Gelman & Pardoe, 2006) of the model using only the fixed effects coefficients for prediction and then with additional random effects coefficients associated with successively lower levels of nesting. The *R* ^num^ and *R* ^bio^ values submitted to the analysis, along with R code describing the statistical model, are electronically archived (Reum 2018, https://doi.org/10.6084/m9.figshare.7210046.v2).

### 2.3 Community‐wide mean PPMR

Community‐wide mean PPMRs for the EBS, AI, and GoA (i.e., mean PPMR for the sampled communities) were calculated from predator species, size class, and subregion *R* ^bio^ as follows. First, the fitted hierarchical model was used to predict *R* ^bio^ (${\hat{R}}^{\mathrm{bio}}$) across size classes and subregions for each predator species. For clarity, we use the subscripts *a*, *b*, *c,* and *d* to index size class‐, species‐, region‐, and subregion‐specific estimates. Second, for each size class and predator species, region‐level estimates ($R_{a,b,c}^{\mathrm{bio}}$) were obtained through weighted averaging of subregion‐level predicted values. For *d* = 1, … *y* subregions, $R_{a,b,c}^{\mathrm{bio}}$ was calculated following:

(7) $$R_{a,b,c}^{\mathrm{bio}}=\sum_{d=1}^{y}{\hat{R}}_{a,b,c,d}^{\mathrm{bio}}\times q_{a,b,c,d}$$

where *q_a_*_,_ *_b_*_,_ *_c_*_,_ *_d_* represent the proportional contribution of predator species to total community biomass within a given size class, region, and subregion:

(8) $$q_{a,b,c,d}=\frac{B_{a,b,c,d}}{\sum_{d=1}^{y}B_{a,b,c,d}}$$

*B* is the time‐averaged biomass density (kg/km) for a given body mass class, predator species, region, and subregion based on bottom trawl survey data. Time‐averaged biomass densities were used in the calculation because diet data were also pooled across years.

Third, for *b* = 1, … *z* predator species, community‐wide *R* ^bio^ estimates for each predator size class and region ($R_{a,c}^{\mathrm{bio}}$) were calculated as follows:

(9) $$R_{a,c}^{\mathrm{bio}}=\sum_{b=1}^{z}R_{a,b,c}^{\mathrm{bio}}\times q_{a,b,c}$$

where *q_a,b,c_* is the proportional contribution of predator species to total community biomass within a given size class and region:

(10) $$q_{a,b,c}=\frac{B_{a,b,c}}{\sum_{b=1}^{z}B_{a,b,c}}$$

The community‐wide mean *R* ^bio^ was resolved for each predator body mass class to evaluate potential size‐dependent nonlinearities. If insufficiency of diet data precluded subregion‐specific predictions (that is, ${\hat{R}}_{a,b,c,d}^{\mathrm{bio}}$ in Equation (7)), then predictions generated for the region were used instead (${\hat{R}}_{a,b,c}^{\mathrm{bio}}$). If region‐level predictions were not feasible, then predictions were generated for the species (${\hat{R}}_{a,b}^{\mathrm{bio}}$). Overall, subregion to species‐level predicted *R* ^bio^ values were estimated for 90% to 95% of the fish biomass in each region. For the remaining 5% to 10% of fish biomass, family‐level *R* ^bio^ was estimated. Uncertainty described by the posterior distributions from the hierarchical model was propagated to the community‐wide mean *R* ^bio^ estimates. For comparative purposes, we repeated the preceding methods with the *R* ^num^ hierarchical model.

## 3 RESULTS

**Figure 1:**
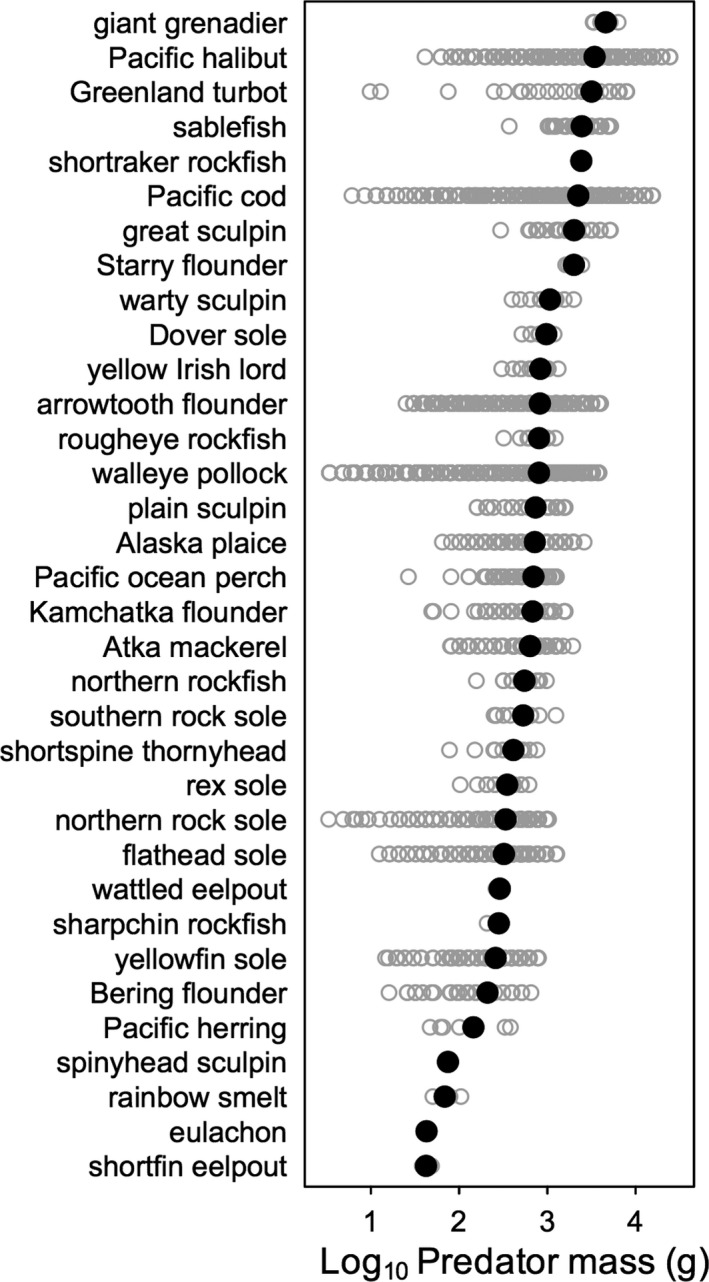
Overview of body masses of individual fish predators sampled from Alaskan marine ecosystems. Open gray circles: body mass of individual predators; black closed circles: mean body mass of all individual predators

**Figure 2:**
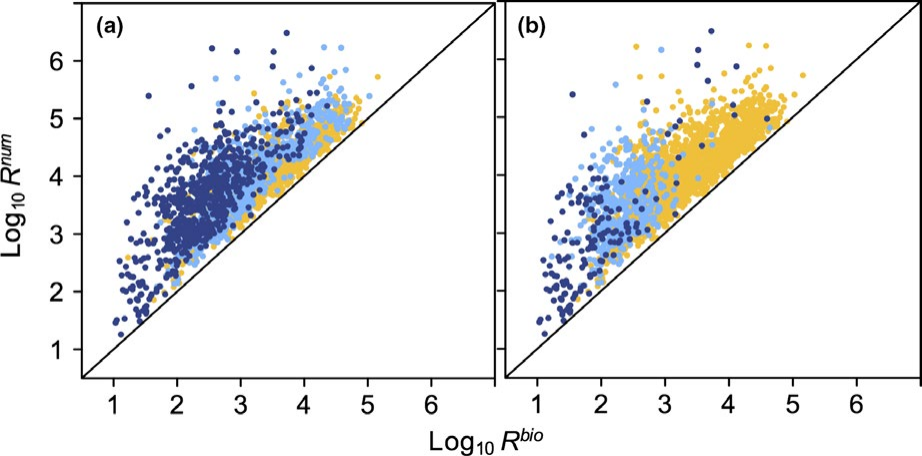
Comparison of *R* ^bio^ and *R* ^num^ for predators within the same body mass class, species, and subregion. Color code corresponds to the proportion of fish in diets by (a) biomass and (b) number: yellow, <10%; light blue 10%–50%; dark blue >50%. Black diagonal line corresponds to the 1:1 line

Diets from 170,689 individual predators were included in the analysis, from 34 fish species in 10 families and six orders (Supporting Information Table S1). Collectively, fish predator body masses spanned ~4 orders of magnitude (Figure 1). *R* ^num^ values were approximately an order of magnitude higher than *R* ^bio^, but individual values were up to four orders of magnitude higher (Figure 2). Low *R* ^bio^ and *R* ^num^ values were generally associated with predator diets containing a high proportion of fish (Figure 2).

For both *R* ^bio^ and *R* ^num^ models, MCMC chains converged, were well‐mixed, and exhibited low autocorrelation (<0.05). Posterior predictive checks and visual inspection of the residuals and fitted values indicated that the data were adequately described by the models.

**Figure 3:**
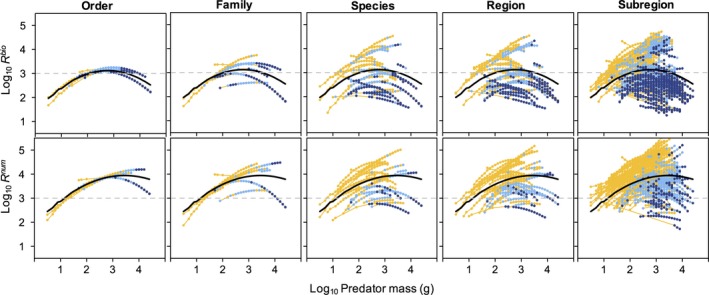
Top panel: Predicted relationships between log_10_ predator body mass and mean *R* ^bio^ for Alaskan marine fish predators according to order, family, species, region, and subregion. Color code corresponds to the average proportion of fish in predator diets by weight: yellow, <10%; light blue, 10%–50%; dark blue >50%. Bottom panel: Predicted relationships between log_10_ predator body mass and mean *R* ^num^. Color code corresponds to the average numerical proportion of fish in predator diets. Black line corresponds to mean body mass relationship (fixed effect). A horizontal dashed gray line is overlaid at *R* ^bio^ and *R* ^num^ = 10^3^ to aid comparisons

Overall, *R* ^bio^ tended to vary with predator body mass in a nonlinear, dome‐shaped manner, but the fixed effect slope coefficients for both the linear (median and 95% HPD credible interval: 0.02 and −0.37 to 0.36) and quadratic (−0.16 and −0.46 to 0.20) predator body mass predictors did not differ from zero (Figure 3). Order‐ and family‐level relationships were relatively similar to the fixed effect relationships, but variation at the species level was substantially greater (Figure 3). Across species and size classes, *R* ^bio^ ranged from approximately 10^1.5^–10^4.5^ (32–31,000) and within a single intermediate predator body mass class (10^2.5^ g) *R* ^bio^ values spanned approximately two orders in magnitude (Figure 3). Species and size classes that fed heavily on fish generally showed the lowest mean *R* ^bio^ values (Figure 3). Linear and quadratic slope coefficients differed from zero for three and seven species, respectively (Supporting Information Figures S1 and S2). The predicted range in species *R* ^bio^ values increased relatively little with inclusion of region‐ and subregion‐level coefficients (Figure 3).

Mean predicted *R* ^num^ was higher than *R* ^bio^ and tended to increase more linearly with predator body mass, but the fixed effect linear (median and 95% HPD credible interval: 0.18 and −0.26 to 0.57) and quadratic slopes (−0.12 and −0.47 to 0.57) also did not differ from zero (Figure 3). Variation in predicted *R* ^num^ was similar to that in *R* ^bio^ at order‐ and family‐levels and species‐level variation was also notably higher, ranging from ~102–10^4.8^ (100–63,000; Figure 3). Linear and quadratic slope coefficients differed from zero for four and six species, respectively (Supporting Information Figures S1 and S2). As for *R* ^bio^, inclusion of region‐ and subregion‐level coefficients only modestly increased the range of predicted values (Figure 3).

**Figure 4:**
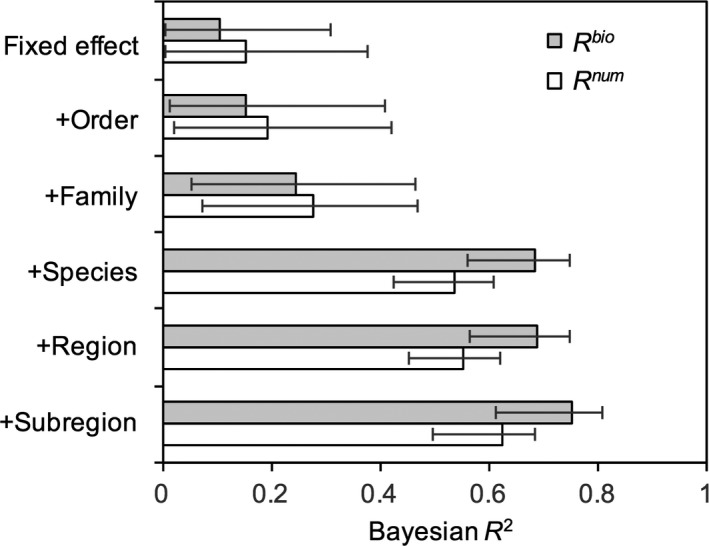
Bayesian explained variance (*R* ^2^) of the model, sequentially adding in higher levels of nested random effects. Error bars indicate the 95% highest posterior density credible intervals

Evaluation of Bayesian *R* ^2^ also highlighted the relative importance of species‐level coefficients in accounting for variation in the data (Figure 4). For *R* ^bio^, the fixed effect “explained” only 10.4% of variance, with inclusion of order‐ and family‐level coefficients increasing this to just 15.2% and 24.6%, respectively. But, at the species level, *R* ^2^ improved substantially to 69.5%. Further including region‐ and subregion‐level coefficients only added another 0.3% and 5.7% to *R* ^2^, respectively. Similar changes in *R* ^2^ with level of analysis were apparent for the *R* ^num^ model (Figure 4).

**Figure 5:**
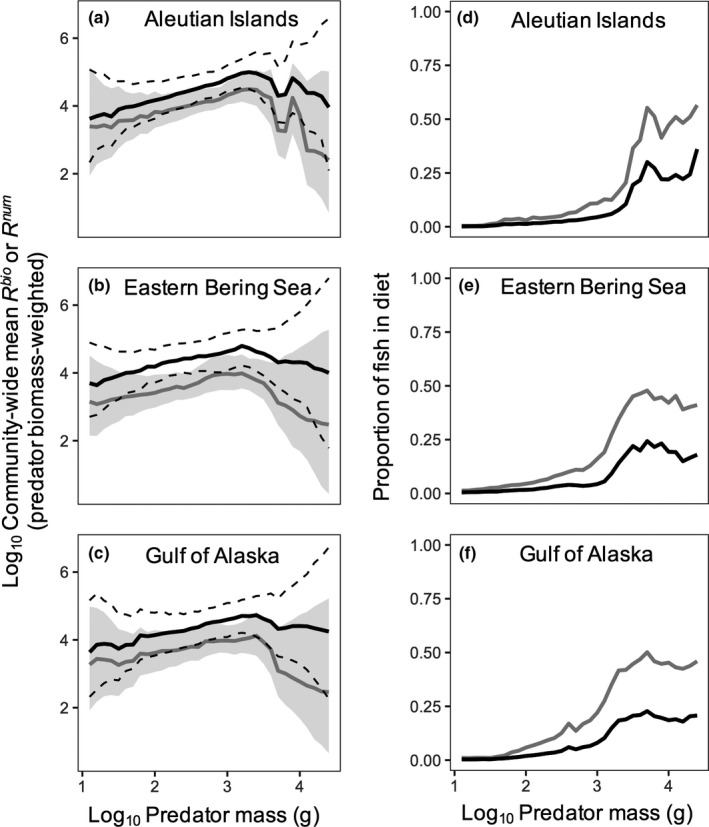
(a–c) Community *R* ^bio^ and *R* ^num ^for Aleutian Islands, Eastern Bering Sea, and Gulf of Alaska. Solid gray lines indicate median *R* ^bio^; gray band indicates the 5th and 95th uncertainty intervals. Black solid line corresponds to median *R* ^num^; dashed lines indicate the 5th and 95 uncertainty intervals. Uncertainty is based on prediction errors from the fitted species and region‐specific *R* ^bio^ or *R* ^num^ body mass relationships. (d–f) Proportional contribution of fish to predator diets at the community level by biomass (gray line) and numbers (black). Diet proportions are weighted according to predator biomass

Relationships between community‐wide mean *R* ^bio^ and predator body mass were slightly domed shaped in all three regions, with peak values occurring at a predator body mass near 10^3^ g (Figure 5a–c). The decrease in community‐wide mean *R* ^bio^ at larger predator sizes coincided with higher proportions of fish (>0.20) in predator diets (Figure 5d–f). Uncertainty in the community‐wide *R* ^bio^ increased toward the upper and lower extremes of the predator body size ranges (Figure 5a–c), partly because of higher prediction uncertainty resulting from lower data coverage at the predator body mass extremes. Typically, median community‐wide mean *R* ^num^ was 0.5–1 order of magnitude higher than community‐wide mean *R* ^bio^ across regions, but showed a similar curvilinear pattern (Figure 5a–c).

## 4 DISCUSSION

The metrics *R* ^bio^ and *R* ^num^ provide complementary insights into predator–prey interactions and the PPMR. Results show that *R* ^bio^ < *R* ^num^, consistent with the numeric dominance of small prey in predator diets, and implying that the metrics are not substitutable. When developing models based on bulk energy flux or comparing diet and stable isotope‐based measures of realized PPMR, *R* ^bio^ will be a more appropriate measure of PPMR. When calibrating preference PPMRs in dynamic models, then *R* ^num^ will be a more appropriate measure of PPMR. For the Alaskan food webs, community‐wide mean *R* ^num^ exceeded *R* ^bio^ by 0.5–1 orders of magnitude. Consequently, equilibrium predictions of food chain length and the unexploited size spectrum slope (e.g., Jennings & Blanchard, 2004; Jennings & Mackinson, 2003) will be under and over‐estimated, respectively, if community‐wide mean *R* ^num^ is used in place of *R* ^bio^. For instance, selecting a PPMR of 10^4^ instead of 10^3^ increases the predicted unexploited biomass of large (10^4^–10^4.1^ g) relative to small (10–10^1.1^ g) predators by ~12% and decreases the predicted relative trophic level of 10 kg predators by 0.25 (Jennings & Blanchard, 2004; Reum et al., 2015). The consistent difference in *R* ^bio^ and *R* ^num^ at multiple levels of aggregation underscore the need to select the form that best matches how PPMR is conceptualized within a particular size‐based modeling framework.

Both *R* ^bio^ and *R* ^num^ were related to predator body mass and variation emerged primarily at the species level. This suggests that individuals of the same size are likely not always interchangeable across species and that changes in species composition will modify community‐wide mean PPMR. From an exploratory perspective, this further suggests that predator traits expressed primarily at the species level (e.g., habitat preferences, morphology, foraging behavior), rather than Family or Order, are likely to have the largest influence on prey selection patterns and thus PPMR. In contrast, region and subregion explained substantially less variation in PPMR, which indicates spatially structured variables that relate prey availability or vulnerability (e.g., temperature, benthic substrate type) may have only a relatively minor influence on PPMR, at least over the spatial scales considered in the analysis. Low relative variation in PPMR over space suggests species may be usefully aggregated into functional groups partly based on PPMR (e.g., Hahm & Langton, 1984, Hansen, Bjornsen, & Hansen, 1994) for the purpose of developing dynamic trait‐based or functional size spectrum models of these systems (Andersen et al., 2016; Blanchard et al., 2017).

A strength of this study is that PPMR was defined for regionally discrete communities which would also be defined as communities for developing size spectrum models. A general prediction of dynamic size spectrum models is that community‐wide mean *R* ^num^ (or *R* ^bio^) will vary with predator body mass in a nonlinear manner over body mass ranges of approximately three to four orders of magnitude but exhibit an overall increasing trend over larger ranges (Hartvig et al., 2011). Interestingly, a roughly dome‐shaped relationship emerged in all three regions for predators spanning approximately four orders of magnitude, similar to observations in one other system (Reum & Hunsicker, 2012), which lends support to these predictions. However, it is unclear to what extent the trends observed here can be extrapolated or are influenced by the predator sizes and species included in the analysis. In stable isotope studies, nonlinear relationships between community‐wide mean *R* ^bio^ and body size are implied by nonlinear body size–trophic level relationships, and this has been observed in at least one plankton food web (Chang et al., 2014). Despite the conceptual consistency with diet‐based estimates of community‐wide mean *R* ^bio^, nonlinear relationships from fish‐dominated communities have not been apparent in stable isotope data (e.g., Reum et al., 2015), although the statistical power to resolve subtle nonlinearity may be low given other sources of uncertainty (Reum et al., 2015). In general, trends in PPMR with body mass and their spatial and temporal ubiquity in food webs are not well understood and are perhaps best addressed by a study that applies a number of the available techniques to the same community over the same time‐period.

For dynamic size spectrum models, direct estimation of the preferred PPMR of predators requires information on diet composition as well as the size composition and abundance of encountered prey (Hartvig et al., 2011; Tsai et al., 2016). Given the complexities of estimating the latter quantities in the field, preferred PPMR is measured more precisely in experiments (e.g., Ursin, 1973), but these closed environments are likely to introduce artifacts (e.g., poor representation of predator and prey refuges, effects of changing light quality and turbidity, etc.). Moreover, such experiments would need to be conducted with many species and body size classes to provide preference functions which could realistically be applied to communities. A more feasible approach might entail estimating preferred PPMR parameters within size spectrum models. To calibrate multispecies size spectrum models to real food webs, parameters controlling the scaling of species abundances are estimated using biomass data (Blanchard et al., 2014), and the approach could be easily extended to estimate preferred PPMR by fitting to *R* ^num^ data as well. Such estimates, however, may have potential biases based on how well *R* ^num^ values derived from stomach content data accurately represent the average prey composition of predators.

Similar to other analyses of PPMR based on stomach contents (e.g., Brose, Jonsson, et al., 2006, Barnes et al., 2010), our study has important caveats. Our analysis used prey collected from stomach samples that in some instances were partially digested, potentially upwardly biasing estimates of *R* ^bio^ and *R* ^num^. We attempted to minimize the level of bias by estimating undigested prey masses with length data when possible and limiting analysis to prey that were largely intact (>75%). In addition, we assumed that the relative abundances of differently sized prey in predator stomachs are proportional to the rates at which they are consumed. If digestion rates are slower for large‐bodied prey compared to small‐bodied prey, they may be overrepresented in the diet data, artificially lowering both *R* ^bio^ and *R* ^num^. Prey digestion rates may also vary by prey type and body composition, but in the absence of information on species‐specific prey digestion rates it is difficult to identify the magnitude of these error sources. While nitrogen stable isotope estimates of community‐wide mean *R* ^bio^ can avoid some of these issues by integrating assimilated prey over longer time periods, they are also sensitive to assumptions regarding the trophic fractionation of nitrogen stable isotopes (Jennings, 2005; Reum et al., 2015). Finally, our analysis was limited to fish predators which are gape‐limited and that spanned ~4 orders of magnitude in body mass. The PPMR patterns observed for this groups may not be indicative of patterns in other taxonomic groups or body size classes.

Empirical estimates of community‐wide mean *R* ^bio^ are needed to parameterize size‐based food web models when realized PPMR is an input (e.g., Borgmann, 1987; Jennings & Blanchard, 2004; Jennings & Mackinson, 2003), and *R* ^num^ is needed to calibrate or test model predictions in cases where preferred PPMR is an input (e.g., Blanchard et al., 2014; Hartvig et al., 2011). Our analysis adds to a body of work that has sought to clarify how PPMR is defined within different size‐ and species‐based food web modeling paradigms (e.g., Gilljam et al. 2011; Nakazawa, 2015;Nakazawa, 2017; Nakazawa et al., 2011; Tsai et al., 2016) and highlights the need to collect and aggregate empirical diet data at appropriate scales with regard to size spectrum theory. The quality and size of our data set (>10^6^ individual predators) is among the largest available for any region, and a future research priority is to develop predictive models of *R* ^bio^ and *R* ^num^ based on predator traits such as feeding mode, morphology, or habitat preference (e.g., Gravel, Poisot, Albouy, Velez, & Mouillot, 2013). Given the intensive sampling required to assemble diet data sets, such models would be particularly valuable for parameterizing multispecies size spectrum models in data‐poor systems. That said, the implications of species‐level variation in PPMR for community structure, productivity, and system responses to pressures such as fishing are only beginning to be explored (e.g., Law et al., 2016) and also warrant further study.

## AUTHORS’ CONTRIBUTION

JCPR and JLB conceived the ideas and designed methodology. JCPR, KH, and KA analyzed the data. JCPR, JS, and JLB led the writing of the manuscript.

## DATA ACCESSIBILITY

Raw diet data for fish predators were obtained from the NOAA Alaska Fisheries Science Center's Groundfish Trophic Interaction Database (https://access.afsc.noaa.gov/REEM/WebDietData/DietDataIntro.php). Species biomasses and length frequency data were provided by the Alaska Fisheries Science Center's Alaska Fisheries Science Center's Resource Assessment and Conservation Engineering Division (https://www.afsc.noaa.gov/RACE/groundfish/survey_data/data.htm). The *R* ^num^ and *R* ^bio^ values, body mass predictor variables, and the taxonomic levels of the predators included in hierarchical modeling analysis are archived electronically (Reum 2018, https://doi.org/10.6084/m9.figshare.7210046.v2).
