# Supplementary material for: Energetically relevant predator–prey body mass ratios and their relationship with predator body size
Source: Ecol Evol. 2018 Dec 27;9(1):201–11. doi: 10.1002/ece3.4715 (PMC6342185; doi:10.1002/ece3.4715)
Supplement: Supplementary file 1 [file ECE3-9-201-s001.docx]

**Fig. S1**. Estimates of species-level coefficients corresponding to the linear term for the *R^bio^* and *R_num_* models. Error bars correspond to the 95% Highest-posterior density (HPD) credible interval. Points correspond to the posterior median.

**Fig. S2**. Estimates of species-level coefficients corresponding to the quadratic term for the *R^bio^* and *R^num^* models. Error bars correspond to the 95% Highest-posterior density (HPD) credible interval. Points correspond to the posterior median.
