# Supplementary material for: Energetically relevant predator–prey body mass ratios and their relationship with predator body size
Source: Ecol Evol. 2018 Dec 27;9(1):201–11. doi: 10.1002/ece3.4715 (PMC6342185; doi:10.1002/ece3.4715)
Supplement: Supplementary file 2 [file ECE3-9-201-s002.docx]

**Table S1**. Taxonomy of fish predators sampled from Alaskan marine ecosystems and the total number of individual predators (prey) from three marine ecosystems. The total prey count also includes estimated numbers based on biomass and mean body mass estimates. AI: Aleutian Islands; EBS, Eastern Bering Sea; GoA, Gulf of Alaska. Common name abbreviations: AT, arrowtooth. Superscripts numbers on family denote Order: Gadiformes, 1; Perciformes, 2; Clupeiformes, 3; Osmeriformes, 4; Scorpaeniformes, 5; and Pleuronectiformes, 6.

| Family | Species | Common name | AI | EBS | GoA |
| --- | --- | --- | --- | --- | --- |
| Gadidae^1^ | *Gadus macrocephalus* | Pacific cod | 3,993 (31,941) | 46,041 (478,165) | 8,949 (137,549) |
|  | *Theragra chalcogramma* | walleye pollock | 2,940 (289,981) | 45,464 (5,050,816) | 6,933 (741,926) |
| Macrouridae^1^ | *Albatrosia pectoralis* | giant grenadier | 82 (822) |  | 61 (131) |
| Zoarcidae^2^ | *Lycodes brevipes* | shortfin eelpout |  | 26 (126) |  |
|  | *Lycodes palearis* | wattled eelpout |  | 36 (372) |  |
| Clupeidae^3^ | *Clupea pallasi* | Pacific herring |  | 96 (13,928) |  |
| Osmeridae^4^ | *Osmerus mordax* | rainbow smelt |  | 73 (873) |  |
|  | *Thaleichthys pacificus* | eulachon |  | 10 (48) |  |
| Anoplopomatidae | ^5^*Anoplopoma fimbria* | sablefish | 17 (551) | 189 (1,057) | 1,122 (25,626) |
| Cottoidea^5^ | *Dasycottus setiger* | spinyhead sculpin |  | 10 (15) |  |
|  | *Hemilepidotus jordani* | yellow Irish lord | 104 (2,079) | 83 (390) | 36 (340) |
|  | *Myoxocephalus jaok* | plain sculpin |  | 709 (3,710) |  |
|  | *Myoxocephalus polyacanthocephalus* | great sculpin |  | 350 (2,139) |  |
|  | *Myoxocephalus verrucosus* | warty sculpin |  | 219 (1,768) |  |
| Hexagrammidae^5^ | *Pleurogrammus monopterygius* | Atka mackerel | 2375 (819,638) | 85 (61,375) | 122 (36,049) |
| Scorpaenidae^5^ | *Sebastes aleutianus* | rougheye rockfish |  |  | 142 (974) |
|  | *Sebastes alutus* | Pacific ocean perch | 1203 (55,851) | 195 (9064) | 798 (41,504) |
|  | *Sebastes borealis* | shortraker rockfish | 10 (14) |  |  |
|  | *Sebastes polyspinis* | northern rockfish | 283 (12,609) |  | 31 (7,228) |
|  | *Sebastes zacentrus* | sharpchin rockfish |  |  | 40 (910) |
|  | *Sebastolobus alascanus* | shortspine thornyhead | 63 (253) |  | 94 (208) |
| Pleuronectidae^6^ | *Atheresthes evermanni* | Kamchatka flounder |  | 750 (2,295) |  |
|  | *Atheresthes stomias* | AT flounder | 589 (3,469) | 4,972 (48,298) | 5,485 (51,149) |
|  | *Errex zachirus* | rex sole | 11 (135) | 11 (41) | 180 (2,181) |
|  | *Hippoglossoides elassodon* | flathead sole | 32 (192) | 5,096 (85,894) | 266 (870) |
|  | *Hippoglossoides robustus* | Bering flounder |  | 537 (6,741) |  |
|  | *Hippoglossus stenolepis* | Pacific halibut | 756 (2,181) | 6,850 (39,113) | 5,148 (36,150) |
|  | *Lepidopsetta bilineatus* | southern rock sole |  |  | 164 (1,118) |
|  | *Lepidopsetta polyxystra* | northern rock sole | 252 (4,784) | 4,508 (79,512) | 165 (2,111) |
|  | *Limanda aspera* | yellowfin sole |  | 7,848 (230,900) | 12 (117) |
|  | *Microstomus pacificus* | Dover sole |  |  | 113 (1,671) |
|  | *Platichthys stellatus* | Starry flounder |  | 48 (334) |  |
|  | *Pleuronectes quadrituberculatus* | Alaska plaice |  | 3254 (46,409) |  |
|  | *Reinhardtius hippoglossoides* | Greenland turbot | 46 (72) | 621 (869) |  |
|  |  | Total | 12756 (1,224,572) | 128,081 (6,164,252) | 29861 (1,087,812) |
